# Differences in Health-Related Quality of Life After Gastric Bypass Surgery: a Cross-Sectional Study

**DOI:** 10.1007/s11695-021-05416-8

**Published:** 2021-04-30

**Authors:** Tobias Antonsson, André Wennersten, Kaisa Sörensen, Sara Regnér, Mikael Ekelund

**Affiliations:** 1 Department of Clinical Sciences Malmö, Section for Surgery, Skåne University Hospital, Lund University, Lund, Sweden; 2 Department of Surgery, Clinical Sciences Malmö, Inga Marie Nilssons gata 47, plan 3, SE-205 02 Malmö, Sweden; 3 Department of Surgery, Skane University Hospital, Lund, Malmo Sweden; 4 Clinical Studies Sweden – Forum South, Skåne University Hospital, Lund, Sweden; 5 Department of Clinical Sciences, Family Medicine and Community Medicine, Malmö, Sweden

**Keywords:** Bariatric surgery, Cross-sectional study, Gastric bypass, Health-related quality of life, Obesity surgery, RAND-36, SF-36

## Abstract

**Background:**

Gastric bypass (GBP) is a surgical method with good evidence of sustainable weight loss, reduced obesity-related comorbidities, and improved health-related quality of life (HRQoL). However, long-term data post-GBP is scarce on HRQoL related to other factors than weight loss, such as impact of socio-economic, age, and gender.

**Aim:**

To investigate long-term HRQoL in GBP patients.

**Methods:**

The study was conducted as a cross-sectional study covering 3 to 9 years post-GBP measuring HRQoL using RAND-36. Association to weight loss, time since surgery, gender, educational level, occupation, and age was analyzed. The participants were included on the basis that they had received a GBP that was performed by Region Skåne, the southernmost administrative healthcare region in Sweden. Recruitment to the study was by mail invitation for an online survey.

**Results:**

Of the total population of 5310 persons receiving the questionnaire, 1339 of the 1372 responders fulfilled the inclusion criteria. Those with low educational level, unemployed, persons on sick leave or disability support, and those with less weight loss reported the lowest HRQoL. The longer time since surgery, the lower the HRQoL.

**Conclusion:**

Less weight loss, longer time since GBP, lower educational level, and lower degree of employment all affect HRQoL negatively after GBP surgery.

**Graphical abstract:**

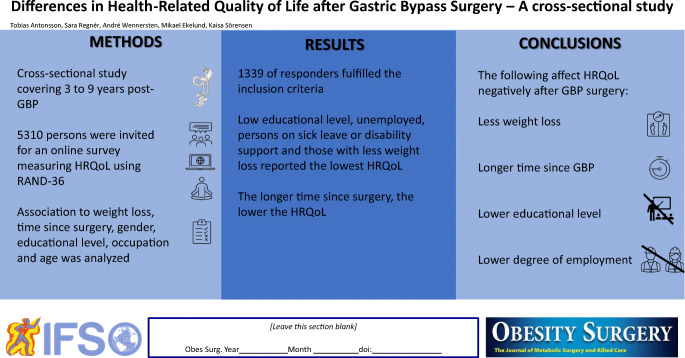

## Introduction

Obesity is a major and increasing public health problem [1, 2]. Quality of life is a multidimensional concept that usually includes subjective evaluations of both positive and negative aspects of life such as physical, mental, and social well-being [3]. Health-related quality of life (HRQoL) encompasses aspects of overall quality of life that can be clearly shown to affect health—either physical or mental. Hence, HRQoL gives an estimate of an individual’s or group’s perceived physical and mental health [4, 5]. In addition to obesity and its comorbidities being serious physical health threats, obese people report poorer HRQoL compared to individuals that has lost weight after GBP surgery [6, 7]. The only currently available treatment providing sustainable and significant weight loss in morbid obesity is bariatric surgery [6, 8]. Both physical and mental health commonly improve upon postoperative weight loss, and there are indications that physical health is most affected [9, 10].

Although GBP surgery has been performed for decades, it was not until the twenty-first century, and the improvement and spread of the laparoscopic technique, that the volumes of GBP surgery markedly increased. Due to the relatively short time of significant surgical volumes, the long-term effects of GBP surgery are not fully understood. In Sweden, about 6000 annual bariatric procedures were performed during the last decade, and laparoscopic GBP has been the dominating bariatric surgical procedure [11]. The hope for improved quality of life may be a strong motivating factor for people deciding to undergo surgery [9]. Patient-reported outcome measures (PROMs), i.e., patients’ self-evaluation of health and well-being, are highly relevant to enable a comprehensive assessment of any given treatment [12].

There is relatively sparse research on how socio-economic factors affect HRQoL after GBP surgery. HRQoL in itself is dependent on many factors and can be used in a complex context that affects the whole individual [13]. The purpose of this study was to explore possible differences in HRQoL post laparoscopic GBP surgery in relation to socio-economic factors, gender, age, weight loss, and years since surgery to get a better knowledge of the patient-reported outcome. This is of importance for healthcare professionals in order to provide balanced information and to increase the possibility of more individualized understanding regarding expectations of HRQoL after surgery.

## Material and Methods

The study was conducted as a cross-sectional study with a quantitative approach. Patients, who have had bariatric surgery during the years 2008–2014, in the geographical and health administrative region of Skåne, Sweden, were selected to participate. Identification of participants was done through the Swedish Obesity Register (SOReg), regional healthcare register, and direct contact with providers in public healthcare and publicly financed private care providers. Exclusion criteria were people living outside the geographical area of Skåne at the time for surgery, deceased persons, or if there was an uncertainty regarding type of surgery performed. All but patients who have undergone GBP surgery were excluded from this study.

Participants were invited, by traditional postal mail, to voluntarily participate in an online survey with questionnaires containing RAND-36, questions about bariatric method and year of operation, pre- and postoperative morphometrics, age, educational level, and employment. The invitation letter was sent out in May 2017 followed by a reminder, to those that had not responded, in August 2017. The web survey was closed in January 2018.

Participation was voluntary, and anonymity was secured by coding. The anonymity was secured by the code key being operated by an independent database collaborator. Data was stored behind double firewalls. Consent to participate, and for data handling, was given by the all participants at completion of the survey.

### RAND-36

The RAND-36 and its commercial version SF-36 are credibly the most widely used health-related quality of life (HRQoL) survey instruments in the world and take 7–10 min to self-administer [14]. The Swedish translation of RAND-36 has shown good reliability regarding test-retest reliability and consistency as measured with Cronbach’s alpha. The value of alpha for the different domains in the instrument ranges from 0.86 to 0.97 [15].

RAND-36 comprised 36 items that assess eight health concepts. Four of the domains are physically oriented, and four of the domains are emotionally and psychosocially oriented. The physical domains consist of physical function (PF) which relates to limitations in physical activity, physical role function (RP) which reflects limitations in daily activities due to physical problems, physical pain (BP), and general health experience (GH). The four psychosocial and emotional domains consist of vitality (VT) that deals with energy and fatigue, social function (SF) that reflects limitations in social activities, and emotional role function (RE) that reflects limitations in daily activities due to emotional problems and mental well-being (MH).

The responses are calculated on a response scale from 0 to 100 for each of the eight domains and 50 points for each domain is a mean norm value of HRQoL for the general population and zero points is equivalent to the worst imaginable and 100 points to the best imaginable results for each domain [16].

### Data Analysis

Data was presented as mean (M) and standard deviation (SD). Non-parametric tests were used in data analysis since the outcome of the variables in the RAND-36 had a negative skew and consisted of ordinal scales. Mann-Whitney *U* test or Kruskal-Wallis test was used when appropriate, and Dunn-Bonferroni post hoc tests were used for multiple comparisons. A *p*-value of <0.05 was considered statistically significant.

Differences in RAND-36 responses were compared related to gender, educational level, (unfinished elementary/compulsory school, upper secondary school, and college/university) and occupation (full-time work, part-time work, parental leave, sick leave/on disability, retired and job seekers). To examine differences in RAND-36 in relation to weight loss, Excess Body Mass Index Loss (% EBMIL) for each participant, using BMI 25 as ideal, was calculated by the formula: (preoperative BMI - postoperative BMI) / (preoperative BMI - 25) × 100. All participants were then divided into four groups according to %EBMIL (<25, 25–<50 %, 50–<75 %, and ≥75%). To examine RAND-36 depending on age, data was divided in quartiles for an even distribution in the number of participants in each age group. To examine whether there are any differences in RAND-36 depending on the time since the surgery, all years (3yrs. through 9 yrs. postoperatively) were compared with all domains.

### Ethical Approval

The study was approved by the Regional Ethical Review Board (Dnr 2016/593). All procedures performed in studies involving human participants were in accordance with the ethical standards of the institutional and/or national research committee and with the 1964 Helsinki declaration and its later amendments or comparable ethical standards.

## Results

A total of 5310 persons, 74% women and 26% men, were invited to the survey and 1372 persons responded. Out of the 1372 persons, 2.4% (*n* = 33) were excluded from the database, because of sleeve gastrectomy surgeries (*n*=20), unable to specify type of surgery (*n*=12), and unlikely negative %EBMIL (*n*=1). Of the participants (*n* = 1339), 79% were women and 21% were men. Mean age at year since surgery was 44 years (range 18 to 67). Mean BMI preoperatively was 43.8±6.1 kg/m^2^, and mean BMI postoperatively was 29.1±5.4 kg/m^2^.

The mean postoperative %EBMIL was 80.2±27.4%. Most had undergone surgery in 2013 (22.2%) and in 2014 (21.1%). The most common level of education was completion of secondary school/high school (53.1%) and college or university (33.8%). Most of the participants had full-time work (55.6%) (Tables 1 and 2).

**Table 1:** RAND-36 domains with abbreviations

PF	RP	BP	GH	VT	SF	RE	MH
Physical function	Physical role function	Bodily pain	General health perceptions	Vitality	Social role functioning	Emotional role functioning	Mental health

**Table 2:** Demography of participants (*n*=1339)

All participants	
Age, mean ± SD	44 ± 11
Gender, quantity (%)	
Women	1058 (79)
Men	281 (21)
Weight, mean ± SD	
Preoperative BMI	43.8 ± 6.1
Postoperative BMI	29.1 ± 5.4
%EBMIL	80.2 ± 27.4
Years since surgery, quantity (%)	
9 yrs.	61 (4.6)
8 yrs.	109 (8.1)
7 yrs.	204 (15.2)
6 yrs.	173 (12.9)
5 yrs.	213 (15.9)
4 yrs.	297 (22.2)
3 yrs.	282 (21.1)
Education, quantity (%)	
Unfinished elementary school/elementary school	175 (13.1)
Secondary school	711 (53.1)
University/college	453 (33.8)
Employment, quantity (%)	
Jobseekers	56 (4.2)
Retired	90 (6.7)
Sick/on disability	191 (14.3)
Parental leave	24 (1.8)
Part-time work	233 (17.4)
Full-time work	745 (55.6)

### HRQoL and Years Since Surgery

**Fig. 1:**
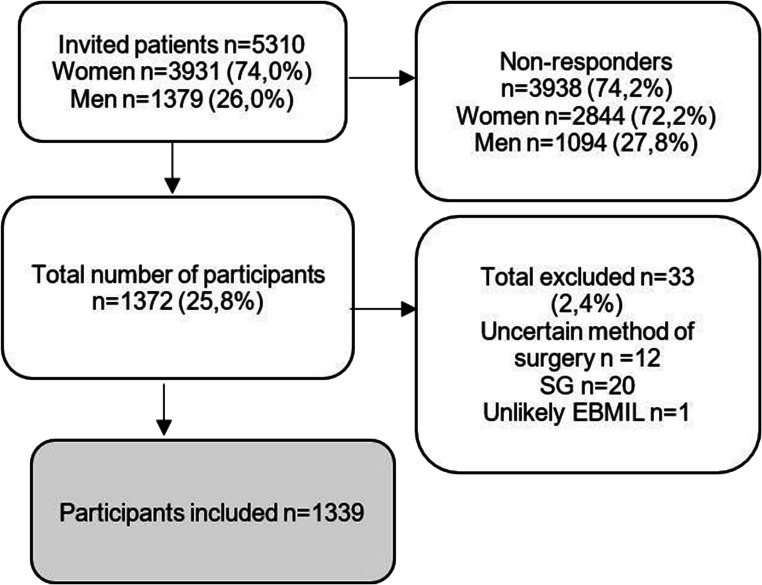
Selection and dropout in database and current study. All having had GBP surgery 2008–2014, in an administratively defined region, were invited for a web-based online survey. GBP, gastric bypass; SG, sleeve gastrectomy; EBMIL, Excessive BMI-loss

**Fig. 2:**
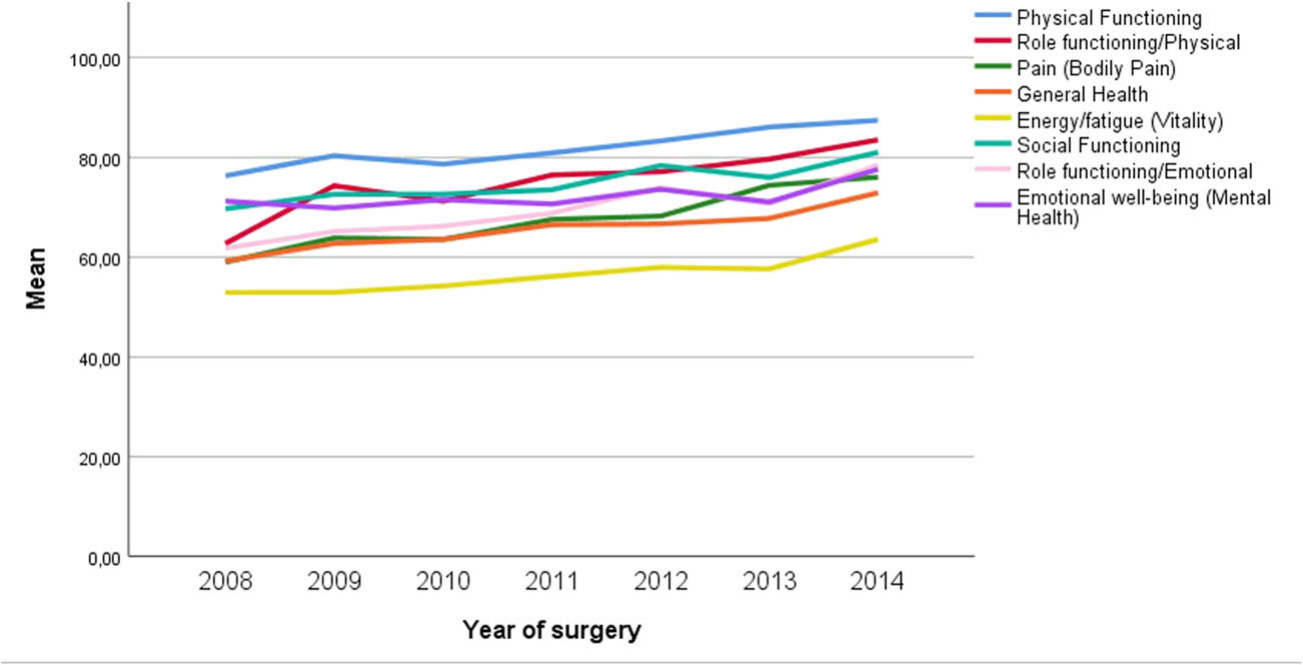
Graph showing decline in all domains of HRQoL in relation to time since surgery

This analysis included all participants who underwent GBP surgery in the range 9 (surgery 2008) to 3 (surgery 2014) years (yrs.) ago. 9yrs. (n = 61), 8 yrs. (n = 109), 7 yrs. (n = 204), 6 yrs. (n = 173), 5 yrs. (n = 213), 4 yrs. (n = 297) and 3 yrs. (n = 282) since surgery. Significant difference (*p* <0.05) was found in the following domains with mostly higher scores from those who underwent surgery more recently: physical function when comparing years since surgery in the groups 9, 8, 7, and 6 with 3 yrs. since surgery; physical role function when comparing 9 with 4 and 3 yrs. since surgery and when comparing 7 and 3 yrs. since surgery; general health experience when comparing 9, 8, and 7 with 3 yrs. since surgery; vitality when comparing 9, 8, 7, and 6 with 3 yrs. since surgery; social function when comparing 9 and 7 with 3 yrs. since surgery; emotional role function when comparing 9 and 7 with 3 yrs. since surgery; mental well-being when comparing 8 with 3 yrs. since surgery; and equally so for the physical pain domain where there were significant differences when comparing 8 and 6 with 3 yrs. since surgery with higher results reported for those that undertook surgery more recently (Figs. 1 and 2).

### HRQoL and Gender

There was a significant difference between men and women for emotional role function with lower scores for women (*p* <0.05). Overall, women stated lower, however non-significant, scores in all domains except physical function and physical role function (Table 3).

**Table 3:** Results in RAND-36 for men and women

RAND-36 domains									
Gender *n*=1339		PF	RP	BP	GH	VT	SF	RE	MH
Women *n*=1058 (79%)	M (SD)	83.4 (22.4)	77.3 (36.5)	69.2 (29.6)	66.5 (25.4)	56.9 (25.8)	75.5 (28.2)	69.9 (40.6)	72.3 (23.1)
Men *n*=281 (21%)	M (SD)	82.5 (24.5)	76.5 (37.0)	71.3 (29.2)	69.2 (23.7)	60.1 (24.1)	78.0 (26.3)	75.2 (38.2)	74.5 (21.1)
	*p*	0.847	0.723	0.238	0.166	0.104	0.270	0.037	0.272

### HRQoL and Level of Education

There were significant differences in RAND-36 between different levels of education (*p* <0.05). Unfinished elementary school/elementary school group had lower scores in RAND-36, while the secondary/high school and university/university groups had higher scores in all domains. In post hoc tests, statistically significant difference was found for unfinished compulsory school/compulsory school compared with both upper secondary school and university/college in the physical domains physical function, physical role function, physical pain, and general health experience and the emotional domains social function and emotional role function (*p* <0.05). There was a significant difference in the emotional well-being of the psychological domain between unfinished compulsory/compulsory school and college/university (*p* <0.05). There was no statistically significant difference between educational levels and the domain vitality (Table 4).

**Table 4:** Results in RAND-36 for educational level

RAND-36 domains									
Education *n*=1339		PF	RP	BP	GH	VT	SF	RE	MH
Unfinished elementary school/elementary school	M (SD)	73.4^a^ (29.0)	64.3^a^ (42.4)	59.2^a^ (32.0)	61.3^a^ (27.6)	53.5 (27.5)	68.5^a^ (31.3)	61.9^a^ (44.3)	67.8^b^ (25.3)
High school *n*=711 (53.1%)	M (SD)	84.0 (21.8)	77.5 (36.0)	69.5 (29.7)	67.5 (25.1)	57.9 (25.5)	77.0 (27.0)	72.5 (39.2)	73.1 (22.6)
University/College n=453 (33,8%)	M (SD)	85.7 (20.5)	81.5 (33.9)	73.9 (27.2)	68.5 (23.8)	58.6 (24.7)	77.3 (27.3)	72.3 (39.5)	74.1 (21.6)
	*p*	<0.001	<0.001	<0.001	0.017	0.093	0.003	0.016	0.030

^a^*p* <0.05 compared to high school and university/college

^b^*p* <0.05 compared to university/college

### HRQoL and Occupation

There were some significant differences when comparing occupation and all domains in RAND-36 (*p* <0.05). The jobseekers and those on sick leave/disability had lower results in all domains of RAND-36 compared to the full-time working group (*p* <0.05). There were statistically significant lower results for part-time work compared to full-time work (*p* <0.05). Those on retirement pension had lower scores in all four physical domains compared to full-time work (*p* <0.05), but no statistically significant difference existed in any of the four emotional domains (Table 5).

**Table 5:** Results in RAND-36 for occupation rate

RAND-36 domains									
Employment *n*=1339		PF	RP	BP	GH	VT	SF	RE	MH
Full-time work *n*=745 (55.6%)	M (SD)	90.6 (15.4)	86.7 (28.1)	78.2 (25.0)	74.2 (21.6)	63.3 (22.9)	84.1 (22.4)	80.4 (34.3)	77.8 (19.4)
Part-time work *n*=233 (17.4%)	M (SD)	84.5^b^ (18.0)	78.6^b^ (35.2)	67.1^b^ (27.6)	65.8^b^ (23.5)	57.4^b^ (24.8)	75.9^b^ (26.3)	71.2^b^ (38.9)	73.0 (22.3)
Parental leave *n*=24 (1.8%)	M (SD)	89.6 (24,4)	90.6 (23.1)	85.3 (21.2)	71.9 (24.1)	54.8 (25.3)	81.3 (23.3)	79.2 (30.8)	78.7 (16.4)
Sick leave/on disability n=191 (14.3%)	M (SD)	59.5^a^ (30.9)	41.6^c^ (43.6)	43.8^d^ (31.9)	44.6^d^ (25.3)	37.6^f^ (24.2)	48.6^d^ (29.1)	36.6^d^ (43.5)	55.4^d^ (25.1)
Retired *n*=90 (6.7%)	M (SD)	72.9^a^ (24.8)	72.8^b^ (38.3)	65.0^e^ (27.6)	66.1^b^ (23.5)	64.1 (23.8)	80.0 (26.0)	79.3 (34.8)	78.4 (18.2)
Job seekers *n*=56 (4.2%)	M (SD)	74.6^a^ (23.8)	65.2^b^ (40.1)	55.4^e^ (30.7)	52.6^a^ (25.6)	40.5^f^ (27.1)	54.5^d^ (29.5)	46.4^d^ (44.8)	52.6^d^ (26.3)
	*p*	<0.001	<0.001	<0.001	<0.001	<0.001	<0.001	<0.001	<0.001

^*a*^*p* <0.05 compared to full-time work, part-time work, and parental leave

^b^*p* <0.05 compared to full-time work

^c^*p* <0.05 compared to full-time work, part-time work, parental leave, job seekers, and retired

^d^*p* <0.05 compared to full-time work, part-time work, parental leave, and retired

^e^*p* <0.05 compared to full-time work and parental leave

^f^*p* <0.05 compared to full-time work, part-time work, and retired

### HRQoL and Weight Loss

In order to compare RAND-36 and weight loss, %EBMIL was used and participants were divided into four groups according to percent lost weight with intervals of 25%. The results showed that the group with %EBMIL <25% had the lowest results in all domains and the group with %EBMIL >75 had the highest mean in all domains. In post hoc tests, significant differences were detected for both %EBMIL <25% and %EBMIL 25–50% compared to %EBMIL >75% with higher results in all domains for the group with the greatest weight loss (*p* <0.05). Also, the group with %EBMIL 50–75% had significantly lower results in all domain (*p* <0.05) except emotional role function compared to those with %EBMIL >75% (Table 6).

**Table 6:** Results in RAND-36 for weight loss (%EBMIL)

RAND-36 domains									
%EBMIL *n*=1339		PF	RP	BP	GH	VT	SF	RE	MH
<25 *n*=44 (3.3%)	M (SD)	54.0^a^ (30.0)	43.2^a^ (42.6)	48.2^a^ (26.5)	44.9^a^ (24.6)	37.4^a^ (25.7)	53.7^a^ (30.2)	52.3^b^ (44.0)	55.6^c^ (25.9)
25–50 n=112 (8.4%)	M (SD)	69.4^a^ (24.0)	62.5^a^ (39.8)	56.3^a^ (27.9)	53.1^a^ (24.0)	47.2^b^ (25.1)	66.3^a^ (27.7)	58.6^b^ (44.8)	68.9^b^ (22.8)
50–75 *n*=372 (27.8%)	M (SD)	80.6^b^ (21.2)	75.9^b^ (36.8)	66.7^b^ (28.2)	64.6^b^ (24.3)	54.4^b^ (24.6)	74.5^b^ (27.9)	68.8 (41.1)	70.1^b^ (22.1)
>75 *n*=811 (60.6%)	M (SD)	87.9 (20.7)	81.6 (34.1)	74.0 (29.3)	71.3 (24.2)	61.6 (24.9)	79.3 (26.8)	74.8 (38.1)	75.5 (22.2)
	*p*	<0.001	<0.001	<0.001	<0.001	<0.001	<0.001	<0.001	<0.001

^a^*p* <0.05 compared with %EBMIL 50–75 and ≥ 75

^b^*p* <0.05 compared with %EBMIL ≥ 75

^c^*p* <0.05 compared with %EBMIL 25–50, 50–75, and ≥ 75

### HRQoL and Age

There were statistically significant differences in all domains (*p* <0.05) except in physical role function and general health experience. In the domain physical function, the group 53–67 years had significantly lower results than all other groups and 46–52 years significantly lower than the group 18–36 years (*p* <0.05). In the domain physical pain, the group 53–67 years had significantly lower results than the group 18–36 years (*p* <0.05). Regarding vitality, the group 18–36 years had significantly lower results than all other groups. Furthermore, the group 37–45 years had statistically lower results than the group 53–67 years (*p* <0.05). In the domains social function, emotional role function, and mental well-being, the group 18–36 years had significantly lower results than all other groups (*p* <0.05). The results for RAND-36 and age are presented in Table 7.

**Table 7:** Results for the age groups in RAND-36

RAND-36 domains									
Age *n*=1339		PF	RP	BP	GH	VT	SF	RE	MH
18–36 years *n*=340 (25.4%)	M (SD)	87.0 (20.8)	80.6 (34.7)	72.7 (29.3)	64.4 (25.0)	51.5 (25.1)	71.3 (29.0)	62.8 (42.2)	68.1 (23.7)
37–45 years *n*=380 (28.4%)	M (SD)	85.1 (22.2)	77.6 (36.6)	71.3 (29.3)	68.3 (25.6)	57.3 (25.9)	77.4 (26.9)	73.0 (39.3)	73.3 (22.4)
46–52 years *n*=305 (22.8%)	M (SD)	82.9 (22.1)	77.5 (35.6)	67.2 (30.2)	67.0 (24.8)	59.4 (25.6)	76.9 (28.1)	72.3 (40.6)	73.5 (23.2)
53–67 years *n*=314 (23.5%)	M (SD)	77.1 (25.0)	72.5 (39.1)	66.7 (29.2)	68.4 (24.7)	62.8 (24.1)	78.6 (26.8)	76.3 (37.3)	76.4 (20.7)
	*p*	<0.001	0.070	0.009	0.084	<0.001	0.002	<0.001	<0.001

## Discussion

The World Health Organization (WHO) defines quality of life as an individual’s subjective perception of their life situation based on the prevailing cultural context and norms in relation to their own goals, expectations, values, and interests [17]. It is a multidimensional and complex concept encompassed by both physical health and mental well-being as well as social relationships, level of independence, and existential issues. Quality of life is thus a subjective experience that may vary over time and is affected by other changes in a life situation than merely the absence or presence of illness [18]. Collecting patient-reported outcome measures (PROMs) is an important way to get first-hand information from patients on the outcome of treatments and surgical procedure they have undergone [19]. The response rate in the present study was about 25% and demonstrates the difficulty in performing follow-up on patients not under active treatment or a direct relation with the caregiver.

In our study, HRQoL, as measured by RAND-36, showed a tendency to decline over time. Previous studies show similar results with improvement in HRQoL between 1 and 3 years post-GBP and then declination at long-term follow-up between 4 and 7 years [6, 20, 21]. This fall in HRQoL may be due to increasing age and age-related illnesses but probably also due to weight regain [6]. In the present study, there was an association between less weight loss and lower HRQoL, indicating poor weight loss and/or weight regain as factors driving the drop in reported HRQoL. Already 2 years post-GBP, fear of weight gain has been shown to resurface, social aspects decline, and self-image is more negative compared to 1 year post-GBP [22]. Although an initial improvement in HRQoL has been shown, studies also indicate that HRQoL can deteriorate in relation to weight gain and after long postoperative follow-up [20, 21]. By qualitative research approaches, it has been stressed that patient education programs and follow-up focusing on changes in daily life are desired [23, 24]. The present study shows differences in HRQoL related to educational level and employment rate. Patients post-GBP with low educational attainment reported lower HRQoL compared to people with higher education. Jobseekers, being on sick-leave, part-time workers, and those on disability support all reported lower HRQoL compared to full-time workers. This is in alignment with a previous study, showing lower self-perceived quality of life in those with low education and low socio-economic levels [25]. Among the general population in Sweden, low level of education and unemployment are social risk factors associated with low HRQoL but also with increased risk of illness and even mortality [1, 26]. There is also a link between high healthcare utilization and low HRQoL [26]. The fact that individuals on sick leave and long-term disability support in the present material reported low HRQoL may also be due to factors related to concurrent illness [27]. Low educational level is also associated with increased risk for hospital admission after GBP surgery [28]. However, inferior HRQoL in these groups may also be due to less %EBMIL which present data indicates when comparing the domains in RAND-36 and weight loss since surgery.

The analysis showed that younger individuals reported better HRQoL in the physical domains compared to older which is comparable with the general population [26]. In contrast in the current study, those of higher age reported better HRQoL in the emotional domains compared to those in the lower age group. There is some evidence indicating that older people generally may appreciate their emotional function higher [29, 30]. Results in current study showed better HRQoL over all in comparison with the general population which may be explained by lower expectations on quality of life resulting in higher HRQoL scoring [31].

The present data shows significant HRQoL gender difference between men and women for emotional role function. The fact that women tended to estimate their HRQoL lower in most of the domains in this study may be substantiated by previous research confirming differences in gender and HRQoL with male GBP patients reporting better HRQoL compared to females [32]. Even in the general population, women report lower HRQoL compared to men, which suggests that present data are comparable to the general population who have not undergone obesity surgery [26, 29].

Previous research on GBP patients has shown a postoperative occurrence of a variety of changes in daily life that needs to be coped. The changes include social relationships, lifestyle and diet, changes in appearance, and changes in self-esteem as a result of weight loss. Not all changes are positive but may instead pose a challenge. In order for these people to receive the right support, healthcare providers need to focus on extensive patient education [24]. From the present study, it is recommended to acknowledge socio-economic factors as important for long-term HRQoL after GBP, and the results may be used to customize follow-up with special attention to younger individuals and those from lower socio-economic backgrounds. There are some limitations in the current study. The study focuses on GBP surgery having been the number one bariatric procedure. During the recent years, sleeve gastrectomy and several other procedures have increased. Post-bariatric HRQoL may differ depending on the procedure performed. Also, as known from other studies and discussed above, long-term independent follow-up is difficult, and a low response rate may provide skewed data and consequently missing information regarding HRQoL in the non-responder group.

In conclusion there are large variations in HRQoL after GBP. Not only less weight loss, weight regain, or time after GBP affect HRQoL negatively but also younger age and socio-economic factors such as low educational level and non-full-time employment. The presented results on patient-reported outcomes may be used to balance healthcare professionals’ and patients’ expectations regarding HRQoL after GBP surgery.
